# Age-period-cohort analysis of gender differential trends in incidence and mortality of non-Hodgkin lymphoma in China, 1990-2019

**DOI:** 10.3389/fonc.2022.1056030

**Published:** 2023-01-06

**Authors:** Yuanyuan Yao, Hui Liu, Fei Zhao, Shengli Zhang, Xingrong Liu

**Affiliations:** ^1^ Department of Social Medicine and Health Management, School of Public Health, Lanzhou University, Lanzhou, China; ^2^ Department of Occupational and Environmental Health, School of Public Health, Lanzhou University, Lanzhou, China; ^3^ Department of Nutrition and Food Hygiene, School of Public Health, Lanzhou University, Lanzhou, China

**Keywords:** NHL, non-Hodgkin lymphoma, incidence, mortality, age-period-cohort model

## Abstract

**Background:**

Non-Hodgkin lymphoma (NHL) is one of the dominant malignancies in the hematological system. This study estimated secular trends in NHL incidence and mortality from 1990 to 2019 and provided comprehensive evaluations of potential age, period, and cohort effects.

**Methods:**

Age-period-cohort (APC) model was used to analyze changes in NHL incidence and mortality with age, period, and birth cohort effects based on data from the Global Burden of Disease Study 2019.

**Results:**

The age-standardized incidence rates (ASIR) of NHL rose by 144.6% and age-standardized mortality rate (ASMR) rose by 27.5% from 1990 to 2019. Local drift for incidence was greater than 0 (p< 0.05) for both genders in each age group. Local drift for mortality rates were greater than 0 (p< 0.05) for males aged 20 to 89 years and females aged 60 to 84 years and less than 0 (p< 0.05) for females aged 20 to 50 years. Period rate ratio (RR) and cohort RR of NHL incidence in China showed an increasing trend, while the trend of male and female mortality was not consistent.

**Conclusions:**

NHL incidence and mortality rates have been increasing in China over the past three decades. Males and older individuals were at high risk for NHL. Thus, attention to the prevention and therapy of NHL would be essential to lessen the disease burden of NHL.

## 1 Introduction

Non-Hodgkin lymphoma (NHL) is one of the dominant malignancies in the hematological system (1), which accounts for about 90% of all lymphoma cases (2). GLOBOCAN 2020 statistics showed that NHL represented 2.8% of total new cases (544,352 new cases) and 2.6% of total deaths (259,793 deaths) (3). Comparing with GLOBOCAN 2012 statistics (4), the number of new cases and deaths in NHL rose by 29.1% and 23.1%, respectively, in 2020.

China, with about 20% of the global population, is experiencing an enormous health burden from NHL (5). The Global Burden of Disease 2019 (GBD 2019) study demonstrated that China accounted for 20.1% of new cases and 17.4% of deaths in NHL around the world (6). A study based on disease surveillance information from the Chinese Center for Disease Control and Prevention showed that mortality from lymphoma and myeloma rose by 4.5% annually between 2004 and 2016 (7). A literature review analysis found that fewer systematic studies of gender and birth cohort differences in NHL incidence and mortality have been reported. In addition, little was known about the factors potentially influencing national trends in NHL incidence and mortality over the last three decades. Thus, this study utilized GBD incidence and mortality data of NHL between 1990 and 2019 to evaluate secular trends of NHL incidence and mortality by decomposing the three effect factors of age, period and cohort with the help of age-period-cohort (APC) model to enrich and broaden the epidemiological understanding of NHL and to offer reference for the development of public health strategies to prevent and treat NHL.

## 2 Methods

### 2.1 Data sources

GBD 2019 covered 204 countries and territories with comprehensive assessment information on health losses from 369 diseases and injuries during the period from 1990 to 2019 (8). Mortality data were obtained from the disease surveillance point system and the cause of death reporting system from the Chinese Center for Disease Control and Prevention. Incidence data were derived from individual cancer registries or aggregated databases, such as “Cancer Incidence in Five Continents (CI5)”. All of the above data were accessed on the GBD online citation tool (http://ghdx.healthdata.org/gbd-results-tool). In this study, we standardized the incidence and mortality of NHL using the global age-standardized population from the GBD 2019 study. International Classification of Diseases (ICD) diagnostic criteria were utilized to stand for NHL (ICD9: 200-200.9, 202-202.98; ICD10: C82-C85.29, C85.7-C86.6, C96-C96.9).

### 2.2 Statistical analysis

APC model was widely used to estimate the effect of three independent factors of age, period and cohort on disease incidence or mortality, and thus to analyze secular trends in disease over time (9). The mathematical expression of the model was: Υ=log*(Μ)*=*μ+αAgei+βPeriodi+γCohorti+ε*

where *M* for the incidence or mortality rate of the corresponding age group, *µ* for the intercept, *αAge*_*i*_ for the effect of the *i* age group (*i*=20-24, 25-29…80-84, 85-89), *βPeriod*_*i*_ for the effect of the *i* period group (*i*=1990-1994, 1995-1999…2010−2014, 2015−2019), *γCohort*_*i*_ for the cohort effect, *ε* for the random error term. The APC model was based on Possion distribution, but there was a high degree of covariance among age, period and cohort in this model, which could not satisfy the independence condition of Possion distribution, so the Intrinsic Estimator (IE) endogenous factor algorithm was introduced to solve the problem of inestimable parameters (10).

APC model estimable parameters included: (1) net drift, which represents the overall annual percentage change; (2) local drift, which indicates the annual percentage change for each age group; (3) longitudinal age curve, which represents fitted longitudinal age-specific rates in the reference cohort after adjusting for period deviations; (4) period (or cohort) rate ratio (RR), which indicates the P period (or C cohort) age-specific RR relative to the reference period (or cohort) group P_0_ (or C_0_) after adjusting for age and period (or cohort) effects (11).

The age range for inclusion in the APC model was 20-89 years, and every 5 years was divided into 1 age group, for a total of 14 groups. Similarly, the diagnostic period was divided into six consecutive time periods from 1990-1994 (median, 1992) to 2015-2019 (median, 2017), and with 2000-2004 as the reference period. The sample consisted of 19 consecutive birth cohorts, which included those born from 1903-1907 (median, 1905) to 1993-1997 (median, 1995), and with 1948-1952 as the reference cohort. The estimated parameters were acquired through the APC web tool available from the National Cancer Institute (12). Wald chi-square tests were used to calculate the significance of the function and estimable parameters. All statistical tests were two-sided, and p<0.05 was considered statistically significant differences.

## 3 Results

### 3.1 Trends in NHL incidence and mortality

**Figure 1:**
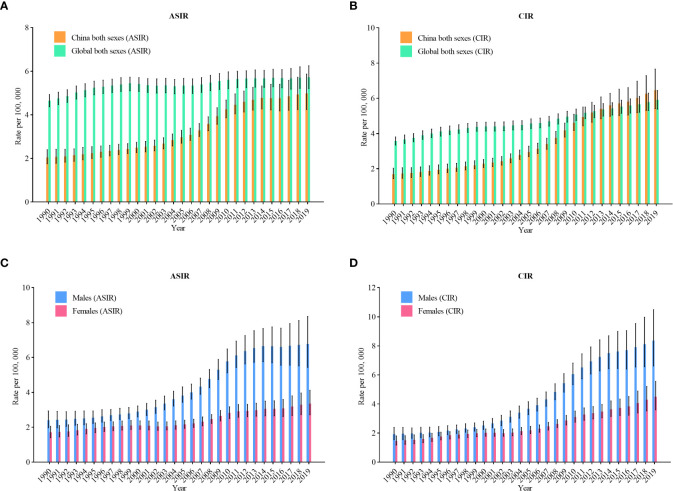
Trends in the age-standardized incidence rates and crude incidence rates (CIR) of non-Hodgkin lymphoma in China from 1990 to 2019. **(A)** ASIR for both genders; **(B)** CIR for both genders; **(C)** ASIR for males and females; **(D)** CIR for males and females.

**Figure 1** shows the overall upward trend of NHL incidence from 1990 to 2019 in China. The age-standardized incidence rates (ASIR) of NHL rose from 2.0 per 100,000 in 1990 to 5.0 per 100,000 in 2019 (an increase of 150%) (**Figure 1A**). In **Figure 1B**, the crude incidence rates (CIR) of NHL rose from 1.7 per 100,000 in 1990 to 6.5 per 100,000 in 2019 (an increase of 282.4%), and China gradually caught up with the global level after 2013. **Figures 1C, D** represents the trends of incidence among various genders. The ASIR for males rose from 2.4 per 100,000 in 1990 to 6.8 per 100,000 in 2019 (an increase of 183.3%), and the CIR rose from 1.9 per 100,000 to 8.4 per 100,000 (an increased of 342.1%). In females, ASIR rose from 1.7 per 100,000 in 1990 to 3.4 per 100,000 in 2019 (an increase of 100%), and CIR rose from 1.5 per 100,000 to 4.5 per 100,000 (an increase of 200%).

**Figure 2:**
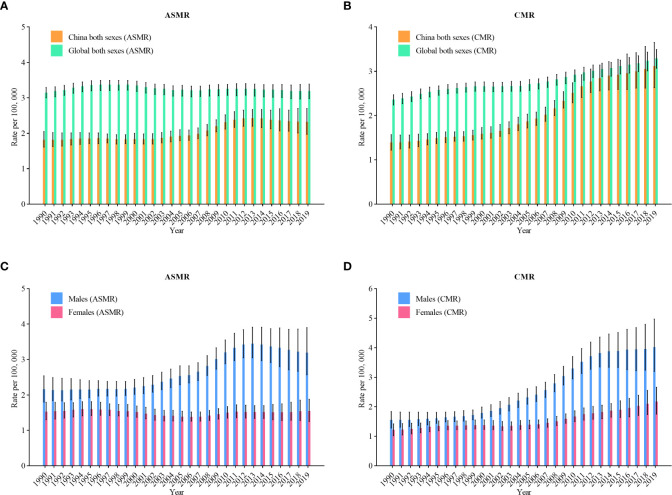
Trends in the age-standardized mortality rates (ASMR) and crude mortality rates(CMR) of non-Hodgkin lymphoma in China from 1990 to 2019. **(A)** ASMR for both genders; **(B)** CMR for both genders; **(C)** ASMR for males and females; **(D)** CMR for males and females.

The trend of mortality in **Figure 2** shows similar trend to **Figure 1**. The age-standardized mortality rates (ASMR) of NHL rose from 1.8 per 100,000 in 1990 to 2.3 per 100,000 in 2019 (an increase of 27.8%) (**Figure 2A**). In **Figure 2B**, the crude mortality rates (CMR) of NHL rose from 1.4 per 100,000 in 1990 to 3.1 per 100,000 in 2019 (an increase of 121.4%). **Figures 2C, D** represents the trends of incidence among various genders. The ASMR for males rose from 2.2 per 100,000 in 1990 to 3.2 per 100,000 in 2019 (an increase of 45.5%), and the CMR rose from 1.6 per 100,000 to 4.0 per 100,000 (an increase of 150%). In females, ASMR rose from 1.5 per 100,000 in 1990 to 1.6 per 100,000 in 2019 (an increase of 6.7%), and CMR rose from 1.2 per 100,000 to 2.2 per 100,000 (an increase of 83.3%).

### 3.2 Net drift and local drift values of NHL

**Figure 3:**
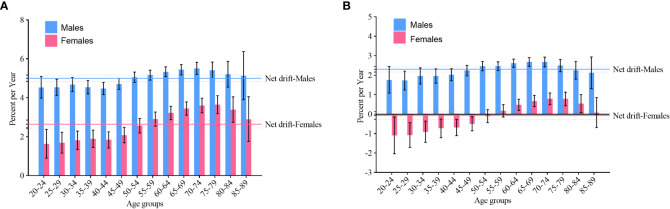
Net drift and local drift values of non-Hodgkin lymphoma by gender in China. **(A)** incidence; **(B)** mortality.

Net drift and local drift values of NHL incidence in China are shown in **Figure 3A**. The net drift values of NHL incidence were 5.0 (95% confidence interval [CI]: 4.8, 5.2) in males and 2.6 (95% CI: 2.4, 2.9) in females. The local drift values were greater than 0 (p< 0.05) for both males and females aged 20-89 years. In addition, we found that the largest local drift value was 5.5 (95% CI: 5.2, 5.8) in males aged 70-74 years and 3.7 (95% CI: 3.2, 4.1) in females aged 75-79 years.

Net drift and local drift values of NHL mortality in China are shown in **Figure 3B**. The net drift values of NHL mortality were 2.3 (95% CI: 2.2, 2.5) and -0.1 (95% CI: -0.3, 0.1) in males and females, respectively. The local drift values for NHL mortality were greater than 0 (p< 0.05) in males aged 20-89 years and in females aged 60-84 years. In addition, we found that the largest local drift value was 2.7 (95% CI: 2.5, 2.9) in males aged 65-69 years and 0.8 (95% CI: 0.5, 1.1) in females aged 70-74 years. The local drift value of NHL mortality among females aged 20-50 years was less than 0 (p< 0.05).

### 3.3 Longitudinal age curves of NHL

**Figure 4:**
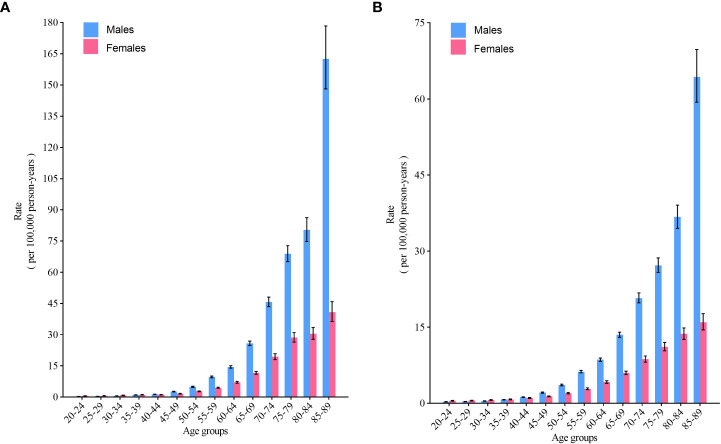
Fitted longitudinal age-specific rates of non-Hodgkin lymphoma and the corresponding 95% CI. **(A)** incidence; **(B)** mortality.

For those younger than 40-44 years, the incidence was similar between males and females among the same birth cohort, whereas for those older than 45 years, the incidence of NHL was significantly greater in males than in females (p< 0.05) and rose dramatically with age (**Figure 4A**). Similar to the trend in incidence, the mortality of NHL was significantly greater in males than in females for those over 45 years of age (p< 0.05) (**Figure 4B**).

### 3.4 Periods and cohort RR of NHL

**Figure 5:**
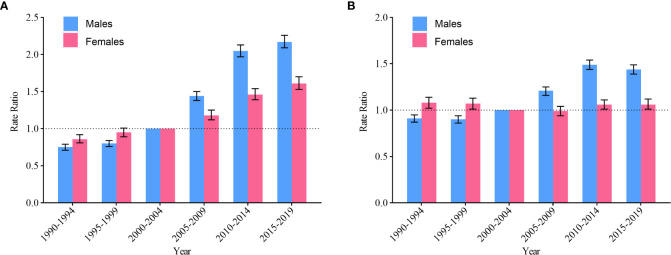
Period RR of non-Hodgkin lymphoma by gender in China. **(A)** incidence; **(B)** mortality.

When adjusted for age and nonlinear period effects, RR was computed in every period versus the reference period (2000–2004), along with the corresponding 95% CI. The period RR of NHL incidence in males and females rose monotonically compared with the reference period (2000–2004) (**Figure 5A**). The period RR of NHL mortality in China, where the period RR of NHL mortality in males showed an overall rising trend but slightly decrease from 2015 to 2019, and the period RR of NHL mortality in females did not change obviously (**Figure 5B**).

**Figure 6:**
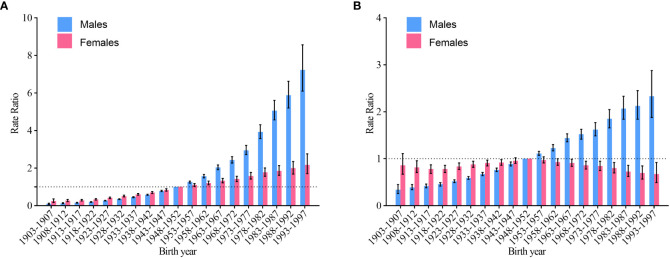
Cohort RR of non-Hodgkin lymphoma by gender in China. **(A)** incidence; **(B)** mortality.

When adjusted for age and nonlinear cohort effects, RR was computed in every cohort versus the reference cohort (1948–1952), along with the corresponding 95% CI. The cohort RR of NHL incidence rose monotonically with age at birth for both males and females, and the rise was more pronounced in the cohort born after 1948-1952 (**Figure 6A**). The cohort RR for males NHL mortality increases with year of birth. In females, the incidence of NHL rose after the 1918-1922 birth cohort, however, this rise stopped after the 1948-1952 birth cohort (**Figure 6B**).

## 4 Discussion

For this study, we estimated secular trends in NHL incidence and mortality in China and provided comprehensive evaluations of potential age, period, and cohort effects. It was found that incidence and mortality of NHL rose in China from 1990 to 2019, and the phenomenon was present for both males and females. It was notable that the CIR of NHL in China gradually caught up with the global level after 2013. Over the past three decades, incidence and mortality has risen for all age groups in males, and females have observed similar trends. Generally, there was monotonic pattern of increasing period and cohort effects, with the risk of NHL incidence and mortality rising as the period and cohort progressed forward.

The burden of disease in NHL varies by age and gender. Overall, the burden of NHL was greater in older individuals and the risk was greater in males than in females. The present study demonstrated that both NHL incidence and mortality rose with age, with the sharpest rate of rise and peak in the 85-89 age group, which was generally consistent with the findings of other studies (13). The phenomenon was partly due to demographic growth and aging. Statistics indicated that the national population rose from 1.14 billion in 1990 to 1.41 billion in 2019 (an increase of 23.7%), and the population aged 65 years and older rose from 63.68 million to 176 million (an increase of 176.4%) (14), with deepening aging and an growing trend of advanced ageing, with the population aged 65 years and older expected to exceed 350 million in 2050 (15). NHL occurs more commonly in the elderly due to the association with the aging process that led to changes in the immune system alterations and the long-term latency of EBV infection (16). Studies have also shown that the burden of disease was greater in males than in females at all ages, which can be partly explained by the association of some risk factors in males, such as exposure to harmful factors like smoking and infections (17). A study concluded that HIV incidence and mortality rates in China increased rapidly from 2004 to 2017 and were greater in males than in females (18). Notably, HIV infection has played an essential role in the etiology of NHL, and it was estimated that approximately 5% to 10% of HIV-infected patients will develop lymphoma (19) and their risk and mortality from NHL increased significantly (20), which may be one of the reasons for the rose disease burden of NHL in our country in recent years. The lower burden of NHL in female patients resembles previous epidemiological studies in that 5- and 10-year survival rates were generally greater in females (21, 22). However, differences in survival were not related to race, ethnicity, year of diagnosis, or NHL subtype (23), which may be related to lifestyle or hormonal differences between genders (24).

Period effects were defined as the risk of incidence or mortality causes by changes in natural conditions or social environment during specific periods of time (12). The overall upward trend of NHL incidence and mortality in China over the period from 1990 to 2019 may be related to the continued advancement of diagnostic and treatment technology for lymphoma (25). As diagnostic technologies for NHL continued to improve, this led to increased detection rates and fewer misdiagnoses and misses, which to some extent can lead to increased incidence. For the elderly, with increasing age and deepening aging of the society, there is no more ideal treatment for NHL so far, although there are advances in treatment technology, and with the more common metastasis and recurrence of NHL, there is still higher mortality rate in the aging society. In 2021, the Chinese Lymphoma Treatment Guidelines (2021 Edition) were published (26), which further improved the standardized diagnosis and treatment of lymphoma in China. Of these, stem cell transplantation and new drugs such as rituximab (27) and immune checkpoint inhibitors (28) may offered a new option for patients with relapsed refractory disease, reflecting to some extent the improved ability to detect subclinical lesions early. Moreover, our increasingly sophisticated universal health information registration system may also be responsible for the increased burden of NHL in recent years. China has been emphasizing health information construction since 1983, and has experienced the evolution of health information, population health information, and universal health information, coordinating regional population health information platforms. The gradual implementation of full coverage of residents’basic health insurance nationwide, the addition of medical information systems in each region, and the establishment and improvement of disease registration and reporting systems have led to wider population coverage, easier disease registration and reporting, and improved incidence and mortality reporting, which could, to some extent, contribute to the increased burden of NHL in China.

Cohort effects were defined as the risk of incidence or mortality for people in the same birth cohort at different ages or for people in different cohorts who received exposure to a factor at the same age (9). Overall, the risk of incidence and mortality has increased progressively with cohort effects in recent years, with greater risk of incidence and mortality for those born later. One possible reason was the change in dietary habits and lifestyle caused by the gradual economic development and the gradual improvement in the standard of living in our country (5). Another reason may be related to the increased social stress faced by the late birth cohort, where chronic stress states can weaken the original anti-tumor environment of immune cells and accelerate tumor progression (29), which to some extent can increase the risk of incidence and death in NHL. Since the potential influences behind each effect were not completely independent, the above two reasons can also be used to explain the period effects. Although China has actively promoted the full coverage of basic medical insurance and urban and rural residents’ medical insurance reform in recent years, the drugs and other treatment options recommended in clinical guidelines were more expensive, and the treatment period of NHL was longer and relapse was more common (30), many NHL patients, especially in less developed provinces or regions in China, were still unable to afford the out-of-pocket portion of the ongoing treatment costs due to lower income levels, higher financial burdens and lack of good medical and health services, and thus were forced to give up treatment, resulting in the phenomenon of poverty due to illness and poverty due to illness, which to some extent reduced the survival rate of these patients and led to an increased risk of death.

There were some limitations in this study. First, although GBD has made several corrections to improve the comparability of data, it still cannot completely avoid bias, such as reporting bias and over-diagnosis bias, which may lead to some bias in the completeness and accuracy of the data. Second, the general limitations described by the GBD collaborative group were all applicable to this study. For example, differences in disease burden between races or ethnicities were not included in the GBD study. Nevertheless, to some degree, racial or ethnic variations have profound impacts on disease incidence and mortality. Finally, the conclusions derived from fitting the APC model inevitably suffer from ecological fallacies when extrapolated to individuals. Therefore, future large-scale individual-based cohort studies should be conducted to confirm the relevant hypotheses presented in our study.

## 5 Conclusion

NHL incidence and mortality rates have been rising in China over the past three decades. In total, the risk of incidence and mortality rises with age and period for both males and females, and the risk of incidence and mortality was consistently greater for males than for females. The cohort effect showed that the earlier the birth, the lower the risk of incidence and mortality, and the later the birth, the greater the risk of incidence and mortality. In this context, it will be necessary to identify risk factors associated with NHL, further develop prevention and therapy guidelines, emphasize screening high-risk persons, and implement measures to enhance the diagnosis and therapy of NHL, and continuously optimize the allocation of public health resources to lessen the burden of NHL in the aging era.

## Data availability statement

The original contributions presented in the study are included in the article/**Supplementary Material**. Further inquiries can be directed to the corresponding author.

## Author contributions

YY conceived the study and wrote the manuscript. XL provided guidance and financial support. HL, FZ and SZ collected and analyzed data. All authors contributed to the article and approved the submitted version.
