# Supplementary material for: Age-period-cohort analysis of gender differential trends in incidence and mortality of non-Hodgkin lymphoma in China, 1990-2019
Source: Front Oncol. 2023 Jan 6;12:1056030. doi: 10.3389/fonc.2022.1056030 (PMC9853163; doi:10.3389/fonc.2022.1056030)
Supplement: Supplementary file 1 [file DataSheet_1.zip › Supplementary Material/TABLE S1-The data collection process.docx]

**TABLE S1** │ The data collection process

Visit website <https://ghdx.healthdata.org/gbd-results-tool.>


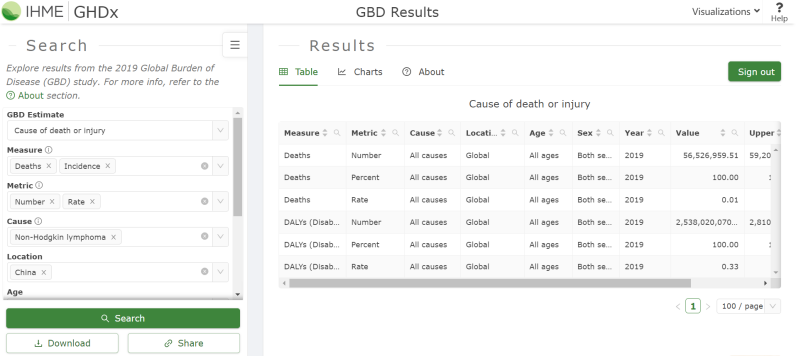


Location = China; Year = select all (from 1990 to 2019); Context = Cause; Age = age-standardized, 20-24, 25-29, 30-34, 35-39, 40-44, 45-49, 50-54, 55-59, 60-64, 65-69, 70-74, 75-79, 80-84, 85-89; Metric = Number and Rate; Measure = Incidence and Deaths; Sex= Both, Female, and Male; Cause = B.1.26 Non-Hodgkin lymphoma.
