# Supplementary material for: Age-period-cohort analysis of gender differential trends in incidence and mortality of non-Hodgkin lymphoma in China, 1990-2019
Source: Front Oncol. 2023 Jan 6;12:1056030. doi: 10.3389/fonc.2022.1056030 (PMC9853163; doi:10.3389/fonc.2022.1056030)
Supplement: Supplementary file 1 [file DataSheet_1.zip › Supplementary Material/TABLE S2-Wald Chi Square tests for estimable functions in the APC model.docx]

**Table S2.** Wald Chi Square tests for estimable functions in the APC model.

| Null Hypothesis | Incidence-M | | Incidence-F | | Mortality-M | | Mortality-F | |
| --- | --- | --- | --- | --- | --- | --- | --- | --- |
|  | Chi-Square | P-Value | Chi-Square | P-Value | Chi-Square | P-Value | Chi-Square | P-Value |
| Net Drift = 0 | 3609.01 | <0.001 | 638.24 | <0.001 | 989.53 | <0.001 | 0.53 | 0.47 |
| All Period RR = 1 | 4509.14 | <0.001 | 794.88 | <0.001 | 1252.88 | <0.001 | 19.03 | 0.002 |
| All Cohort RR = 1 | 4083.32 | <0.001 | 827.68 | <0.001 | 1281.01 | <0.001 | 62.83 | <0.001 |
| All Local Drifts = Net Drift | 36.20 | <0.001 | 64.06 | <0.001 | 24.78 | 0.04 | 54.48 | <0.001 |
