# Supplementary material for: Age-period-cohort analysis of gender differential trends in incidence and mortality of non-Hodgkin lymphoma in China, 1990-2019
Source: Front Oncol. 2023 Jan 6;12:1056030. doi: 10.3389/fonc.2022.1056030 (PMC9853163; doi:10.3389/fonc.2022.1056030)
Supplement: Supplementary file 1 [file DataSheet_1.zip › Supplementary Material/TABLE S3-The local drifts for China by gender.docx]

**Table S3.** The local drifts for China by gender (%)

| Age | Incidence-M | Incidence-F | Mortality-M | Mortality-F |
| --- | --- | --- | --- | --- |
| 22.5 | 4.5 (4.0,5.1) | 1.6 (0.9,2.4) | 1.8 (1.1,2.5) | -1.1 (-2.0,-0.1) |
| 27.5 | 4.5 (4.1,5.0) | 1.7 (1.2,2.2) | 1.7 (1.2,2.2) | -1.1 (-1.7,-0.4) |
| 32.5 | 4.7 (4.3,5.1) | 1.8 (1.3,2.3) | 2.0 (1.6,2.4) | -0.9 (-1.5,-0.4) |
| 37.5 | 4.5 (4.2,4.9) | 1.9 (1.5,2.3) | 2.0 (1.6,2.3) | -0.7 (-1.2,-0.3) |
| 42.5 | 4.5 (4.2,4.8) | 1.8 (1.4,2.3) | 2.0 (1.7,2.3) | -0.7 (-1.1,-0.3) |
| 47.5 | 4.7 (4.4,5.0) | 2.1 (1.7,2.5) | 2.2 (2.0,2.5) | -0.5 (-0.9,-0.1) |
| 52.5 | 5.1 (4.8,5.3) | 2.6 (2.2,2.9) | 2.5 (2.2,2.7) | -0.1 (-0.4,0.2) |
| 57.5 | 5.2 (4.9,5.4) | 2.9 (2.6,3.3) | 2.5 (2.3,2.7) | 0.2 (-0.2,0.5) |
| 62.5 | 5.3 (5.1,5.6) | 3.2 (2.9,3.6) | 2.6 (2.4,2.8) | 0.5 (0.2,0.8) |
| 67.5 | 5.5 (5.2,5.7) | 3.5 (3.1,3.8) | 2.7 (2.5,2.9) | 0.7 (0.4 ,1.0) |
| 72.5 | 5.5 (5.2,5.8) | 3.6 (3.2,4.0) | 2.7 (2.4,2.9) | 0.8 (0.5,1.1) |
| 77.5 | 5.4 (5.0,5.8) | 3.7 (3.2,4.1) | 2.5 (2.2,2.8) | 0.8 (0.4,1.1) |
| 82.5 | 5.2 (3.9,6.4) | 3.4 (2.7,4.1) | 2.3 (1.8,2.7) | 0.5 (0.1,1.0) |
| 87.5 | 5.1 (3.9,6.4) | 2.9 (1.8,4.1) | 2.1 (1.3,2.9) | 0.1 (-0.7,0.9) |
